# Fat Mass and Obesity Associated (*FTO*) Gene and Hepatic Glucose and Lipid Metabolism

**DOI:** 10.3390/nu10111600

**Published:** 2018-11-01

**Authors:** Tooru M. Mizuno

**Affiliations:** Division of Endocrinology and Metabolic Disease, Department of Physiology & Pathophysiology, Max Rady College of Medicine, Rady Faculty of Health Sciences, University of Manitoba, Winnipeg, MB R3E 0J9, Canada; Tooru.Mizuno@umanitoba.ca; Tel.: +1-204-789-3765

**Keywords:** FTO, liver, gluconeogenesis, lipogenesis, glucose, insulin, type 2 diabetes, non-alcoholic fatty liver disease

## Abstract

Common genetic variants of the fat mass and obesity associated (*FTO*) gene are strongly associated with obesity and type 2 diabetes. FTO is ubiquitously expressed. Earlier studies have focused on the role of hypothalamic FTO in the regulation of metabolism. However, recent studies suggest that expression of hepatic FTO is regulated by metabolic signals, such as nutrients and hormones, and altered FTO levels in the liver affect glucose and lipid metabolism. This review outlines recent findings on hepatic FTO in the regulation of metabolism, with particular focus on hepatic glucose and lipid metabolism. It is proposed that abnormal activity of hepatic signaling pathways involving FTO links metabolic impairments such as obesity, type 2 diabetes and nonalcoholic fatty liver disease (NAFLD). Therefore, a better understanding of these pathways may lead to therapeutic approaches to treat these metabolic diseases by targeting hepatic FTO. The overall goal of this review is to place FTO within the context of hepatic regulation of metabolism.

## 1. Introduction

Genetic factors contribute to the susceptibility to metabolic diseases. A genome-wide association study has identified common variants in the fat mass and obesity associated (*FTO*) gene to be associated with obesity and type 2 diabetes [1,2,3]. Individuals homozygous for the risk allele have increased adiposity, fasting blood glucose and hepatic glucose production compared to those homozygous for the low risk allele [1,4]. These associations have been confirmed by replication across diverse ethnic backgrounds [5]. The initial study reported that the significant association of *FTO* variants and type 2 diabetes risk no longer exists after adjusting adiposity [1]. However, subsequent studies demonstrated that *FTO* variants are associated with an increased risk of type 2 diabetes at least partly independent of obesity [4,6,7,8,9,10,11,12]. These findings suggest that FTO is implicated in the regulation of both body weight and glucose metabolism.

Although variants in the *FTO* gene are unequivocally associated with obesity and type 2 diabetes, the biological function of FTO itself is not fully understood. FTO belongs to the superfamily of Fe (II)- and 2-oxoglutarate-dependent dioxygenases and plays a role in demethylation of nucleic acids [13,14,15,16]. It has been shown that transfected FTO localizes to the nucleus [13,17]. These biological properties of FTO suggest the possibility that FTO may regulate the expression of genes through modification of methylation–demethylation states of genes. It is therefore proposed that FTO plays a role in the regulation of metabolism, possibly by altering gene expression in metabolically active tissues.

## 2. Biological Function of FTO

A homozygous loss-of-function mutation of the *FTO* gene in humans causes severe growth retardation and delays the development of the central nervous system without causing any obvious phenotypic changes in metabolism [18]. Heterozygous loss-of-function mutations in the *FTO* gene were found in both lean and obese individuals [19]. Moreover, studies showed that *FTO* mRNA levels are either unchanged in adipose tissue or increased in peripheral blood cells in individuals with *FTO* obesity-risk alleles, compared to individuals having no risk alleles [20,21]. Therefore, it is likely that the obese phenotype in individuals carrying the *FTO* risk alleles is not due to loss-of-function of FTO itself, but rather due to changes in FTO function and/or expression or function of other genes whose expression may be altered by *FTO* variants.

Mice deficient in FTO exhibit severe growth retardation [22]. Deficiency of FTO or partial loss-of-function mutations in the *FTO* gene is associated with reduced adiposity, while enhanced FTO expression results in increased adiposity and body weight in mice [17,22,23]. High-fat diet-induced obesity is attenuated and exaggerated in FTO-deficient mice and transgenic mice overexpressing FTO, respectively [23,24,25]. Contrary to these findings, other studies demonstrated increased fat mass or percent body fat in mice lacking FTO [24,26,27]. These findings suggest that FTO plays a role in the regulation of whole body metabolism in mice. However, the phenotypes observed in these mouse models are complex and are only partially consistent with the clinical phenotypes of human FTO deficiency. Consequently, these findings raise the question whether the role of FTO in the control of metabolism is species-specific. It is also possible that whole body loss-of-function mutations may mask tissue-specific function of FTO in metabolism. To support this possibility, neuron-specific FTO-deficient mice recapitulate metabolic phenotypes of whole-body FTO deficiency, while mice lacking FTO only within the hypothalamus only partially exhibit the metabolic phenotype observed in mice with complete FTO deficiency [24,26]. A limited number of metabolic phenotypes (i.e., body weight, adiposity, food intake and energy expenditure) were examined in these studies and thus it is not known whether FTO plays a role in the regulation of other aspects of metabolism (such as glucose homeostasis) in a tissue-specific manner. Complete absence of FTO improves glucose tolerance and insulin sensitivity in mice, suggesting a possible role of FTO in the regulation of glucose homeostasis [22,26]. Since the liver plays a major role in the regulation of glucose and lipid metabolism, it was assumed that hepatic FTO participates in the regulation of metabolism. Research has just started to uncover the role of hepatic FTO in the regulation of metabolism.

## 3. Regulation of Hepatic FTO Expression by Metabolic Signals

If hepatic FTO plays a role in the regulation of metabolism, expression of FTO in the liver may be altered in response to changes in metabolic state of the body. Hepatic *FTO* mRNA levels are increased by fasting without a significant change in its protein levels and reduced by glucose treatment in mice [24,28]. Consistent with these findings in mice, levels of *FTO* mRNA and protein are increased by fasting and reduced by re-feeding in chickens [29,30]. Levels of gluconeogenic phosphoenolpyruvate carboxykinase 1 (*PCK1*) and glucose-6-phosphatase (*G6PC*) mRNA behave in a similar manner as *FTO* mRNA in response to changes in metabolic state. Fasting also causes parallel changes in hepatic expression of FTO and peroxisome proliferator-activated receptor gamma coactivator 1 alpha (PPARGC1A), a transcriptional coactivator that controls expression of genes important for metabolism, such as gluconeogenesis and fatty acid oxidation [29]. *FTO* mRNA levels significantly and positively correlate with blood glucose, *G6PC* mRNA, or *PCK1* mRNA levels [28]. In agreement with these in vivo data, levels of *FTO* and *G6PC* mRNA and protein are increased in mouse hepatocyte cell line AML12 under nutritionally deprived conditions (i.e., mimicking fasted conditions) compared to nutrient rich conditions (i.e., mimicking normally fed conditions) [31]. Thus, the hepatic *FTO* mRNA expression level is altered in response to changes in metabolic state (e.g., fasting) and changes in blood glucose level may contribute to the fasting-induced increase in hepatic *FTO* gene expression.

**Figure 1:**
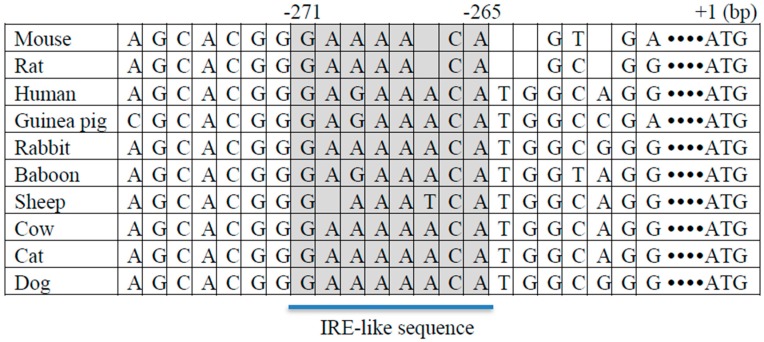
Putative insulin response element (IRE)-like sequence in *FTO* (Fat mass and obesity-associated) promoter. IRE-like sequence GAAAACA was identified in the mouse *FTO* gene promoter (271–265 bp upstream of the transcription start site). The putative IRE with surrounding sequence was aligned in 10 species including, mouse, rat, human, guinea pig, rabbit, baboon, sheep, cow, cat, and dog. The shaded region represents the IRE-like sequence.

Fasting causes alterations in blood level, not only of glucose, but also other metabolites and hormones such as insulin, which plays a role in the regulation of gluconeogenic gene expression. If hepatic FTO plays a role in the regulation of gluconeogenic gene expression, hepatic FTO expression may also be negatively regulated by insulin, and a reduced circulating insulin level may also play a role in mediating the effect of fasting on hepatic FTO expression. Consistent with this assumption, insulin treatment causes reductions in *FTO* and *G6PC* mRNA levels in mouse liver tissues cultured ex vivo and in AML12 cells [31]. Insulin-induced suppression of *PCK1* and *G6PC* expression is mediated via the insulin responsive element (IRE) located within the promoter regions of the *PCK1* and *G6PC* genes [32,33]. Sequence analysis of *FTO* promoter region and alignment analysis showed that the *FTO* gene promoter contains a putative IRE-like sequence across species including mouse and human (Figure 1). Taken together, it is suggested that hepatic *FTO* mRNA expression is negatively regulated by both glucose and insulin, and hepatic FTO regulates gluconeogenic gene expression by mediating the effect of metabolic signals such as nutrients and hormones.

## 4. Hepatic FTO Expression in Obesity and Diabetes

The effect of obesity and diabetes on hepatic FTO expression is controversial. Hepatic *FTO* mRNA levels are reduced in obese mice (*agouti* and *ob/ob*), possibly due to a negative influence by hyperglycemia and hyperinsulinemia [31,34]. Expression of *FTO* mRNA and protein is not altered in livers of mice fed a high-fat diet for 14–17 weeks, while hepatic *FTO* mRNA and protein levels are elevated in mice and rats fed a high-fat diet for 6–12 weeks [24,34,35,36]. In humans, levels of *FTO* mRNA and protein are elevated in the liver of nonalcoholic fatty liver disease (NAFLD) patients who are also hyperglycemic and hyperinsulinemic compared to healthy control subjects [37]. Species difference and duration of high-fat diet feeding may account for these inconsistent findings. Additional studies are needed to fully understand the effect of obesity/diabetes on hepatic FTO expression. Moreover, future studies should investigate the relationship between hepatic FTO expression and possible nutrient and hormonal signals that regulate FTO expression (e.g., glucose and insulin) and the response of hepatic FTO expression to these signals in obesity and diabetes.

## 5. Hepatic FTO and Glucose Metabolism

Changes in *FTO* mRNA expression coincide with changes in the expression level of gluconeogenic genes *PCK1* and *G6PC* mRNA, suggesting the possibility that FTO positively regulates expression of gluconeogenic genes in the liver. Recent studies support this assumption, by demonstrating that enhanced FTO expression stimulates gluconeogenic gene and protein expression in chicken embryonic fibroblast DF-1 cells, human hepatic HuH7 cells and mouse hepatic AML12 cells [29,31,38,39]. Increased FTO level is associated with a higher concentration of lactic acid in culture medium, indicating increased availability of the substrate for gluconeogenesis [39]. Lack of FTO reverses hyperglycemia and improves glucose tolerance in normal and obese/diabetic mice [22,26,40]. Liver-specific FTO overexpression results in increased fasting glucose and insulin levels and impaired glucose tolerance in mice [38]. Collectively, these data suggest that hepatic FTO participates in the regulation of whole body glucose homeostasis at least partly through the regulation of hepatic gluconeogenic gene expression.

**Figure 2:**
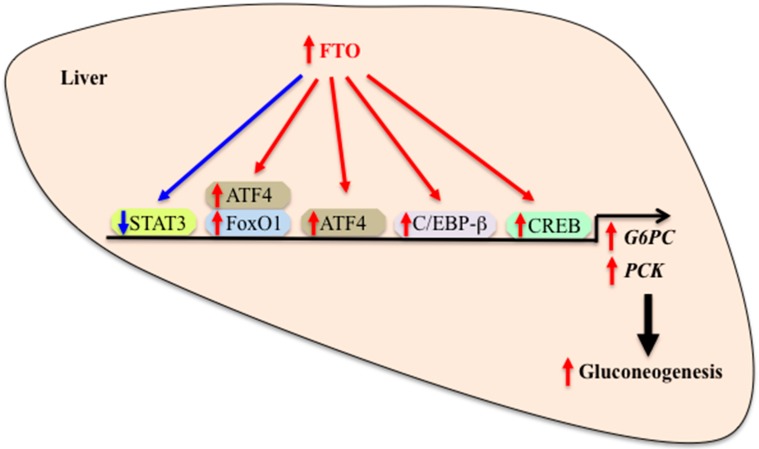
Role of FTO in the regulation of hepatic gluconeogenesis. FTO regulates hepatic gluconeogenic gene expression by altering the activity of and interaction with transcription factors. Increased FTO expression and/or activity causes an increased transcription of genes encoding gluconeogenic enzymes, leading to an increased gluconeogenesis, while reduced FTO expression and/or activity causes the opposite effect. FTO: Fat mass and obesity-associated, G6PC: Glucose-6-phosphatase, PCK: Phosphoenolpyruvate carboxykinase, STAT3: Signal transducers and activators of transcription 3, CREB: cAMP responsive element binding protein, C/EBP-β: CCAAT/enhancer-binding protein-beta, ATF4: Activating transcription factor 4, FoxO1: Forkhead box protein O1. Red arrow: Stimulation. Blue arrow: Inhibition.

What is the possible mechanism by which hepatic FTO regulates gluconeogenic gene expression? Activation of the adipocyte hormone leptin-signal transducers and activators of transcription 3 (STAT3) signaling leads to a reduction of *G6PC* gene expression. Leptin-induced phosphorylation of STAT3 and repression of *G6PC* expression are abolished in FTO-overexpressing human hepatic HuH7 cells [38]. Moreover, enhanced FTO expression in mouse liver causes reduced nuclear translocation of phosphorylated STAT3 and increased *G6PC* mRNA expression. These changes are associated with increased fasting glucose levels and glucose intolerance [38]. These findings suggest that FTO may regulate gluconeogenic gene expression at least partly by blocking the inhibitory effect of leptin-STAT3 signaling on *G6PC* gene expression. The cAMP responsive element binding protein (CREB) plays an essential role in the regulation of *G6PC* transcription in the liver and FTO acts as a transcriptional co-activator of CCAAT/enhancer-binding protein-beta (C/EBP-β) [41]. Enhanced FTO expression also increases expression levels of transcription factors C/EBP-β and CREB1 and increases their binding to the promoter of *G6PC* gene [39]. Additionally, the association of FTO and C/EBP-β is increased in FTO-overexpressing cells. Thus, it is likely that FTO participates in the regulation of gluconeogenic gene expression by altering the activity of and interaction with transcription factors such as STAT3 and C/EBP-β (Figure 2).

FTO positively regulates another transcription factor activating transcription factor 4 (ATF4) that belongs to the family of basic zipper-containing proteins. ATF4 is a positive regulator of gluconeogenic *G6PC* and *PCK1* gene expression and increases glucose production in primary mouse hepatocytes [42]. Levels of ATF4 protein are elevated in the liver of liver-specific FTO transgenic mice [43]. ATL4 deficiency results in improved glucose tolerance, increased insulin sensitivity and reduced glucose production after pyruvate challenge in mice [44,45,46,47]. Levels of gluconeogenic genes are reduced in the liver of ATF4-deficient mice and this effect may be mediated by hepatic ATF4 and/or extra-hepatic (i.e., osteoblastic) ATF4 [42,45]. These data support the possibility that hepatic ATF4 mediates the effect of FTO on glucose metabolism by modulating gluconeogenesis. Regulation of mitochondrial *PCK2* expression requires ATF4 binding to the *PCK2* promoter [48]. Additionally, interaction between ATF4 and forkhead box protein O1 (FoxO1), a major transcription factor for modulating hepatic gluconeogenesis, affects expression of hepatic gluconeogenic gene expression and whole body glucose homeostasis [49]. These data lead to the speculation that ATF4 mediates the stimulatory effect of FTO on hepatic gluconeogenesis by up-regulating gluconeogenic genes through its direct binding to the promoter of these genes and interaction with FoxO1 (Figure 2). Overall, these findings support the role for hepatic FTO in the regulation of gluconeogenesis through the transcriptional regulation of gluconeogenic gene expression. Since FTO functions as a demethylase, it remains possible that FTO plays a role in the regulation of gluconeogenic gene expression at the post-transcriptional and translational levels.

## 6. Hepatic FTO and Lipid Metabolism

**Figure 3:**
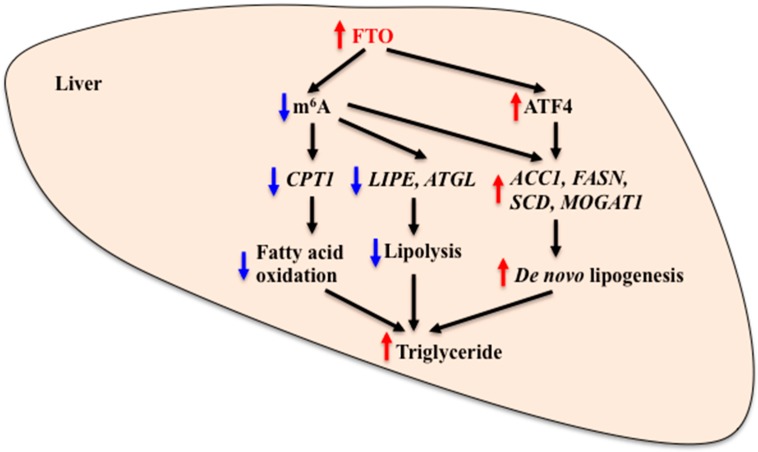
Role of FTO in the regulation of hepatic lipid metabolism. FTO regulates hepatic lipid metabolism by altering the methylation state of genes that are involved in fatty acid oxidation, lipolysis and *de novo* lipogenesis. FTO also regulates hepatic lipid metabolism by altering the activity of transcription factors. Increased FTO expression and/or activity causes a reduction of m^6^A levels and reduces *CPT1*, *LIPE* and *ATGL* mRNA expression, leading to reduced fatty acid oxidation and lipolysis. It also causes an increase in ATF4 expression, which then stimulates expression of lipogenic genes, leading to increased *de novo* lipogenesis in the liver. Reduced FTO expression and/or activity causes the opposite effect. FTO: Fat mass and obesity-associated, m^6^A: N^6^-methyladenosine, CPT1: Carnitine palmitoyltransferase 1, LIPE: Hormone sensitive lipase, ATGL: Adipose triglyceride lipase, ACC1: Acetyl-CoA carboxylase 1, FASN: Fatty acid synthase, SCD: Stearoyl-CoA desaturase, MOGAT1: Monoacylglycerol O-acyltransferase 1, ATF4: Activating transcription factor 4. Red arrow: Stimulation. Blue arrow: Inhibition.

Recent evidence suggests that hepatic FTO is linked to lipid metabolism. FTO expression is increased in the liver of NAFLD patients and animal models of NAFLD [35,37,50]. Hepatic FTO expression is correlated with expression of genes that are involved in lipid metabolism, such as lipogenesis and fatty acid oxidation in rats [51]. FTO overexpression results in increased lipid accumulation in human hepatic L02 and HepG2 cells [35,52]. Lipopolysaccharide (LPS) induces inflammation and leads to abnormal hepatic lipid metabolism. LPS-induced reduction of carnitine palmitoyltransferase 1 (CPT1) levels and increase of triglyceride accumulation in chicken liver coincide with reduced levels of full length *FTO* (chicken *FTO* splice variant 1, *cFTO1*) and increased levels of truncated *FTO* (chicken *FTO* splice variant 4, *cFTO4*) in the liver nuclear extracts [53]. There is a significant inverse relationship between cFTO4 protein levels and *CPT1* mRNA levels in chicken liver. FTO functions as a demethylase for N^6^-methyladenosine (m^6^A) residues in RNA and both cFTO1 and cFTO4 retain the demethylase activity [29,54]. FTO overexpression in HEK293T cells results in a reduction of m^6^A levels in mRNAs [55]. Levels of m^6^A around the translation start site of the *CPT1* gene are reduced in the liver of LPS-treated chickens. These data suggest that increased FTO levels contribute to the increased hepatic triglyceride accumulation by reducing m^6^A levels in *CPT1* mRNA and reducing fatty acid oxidation in the liver. High-fat diet feeding causes increases in the expression of lipogenic genes (acetyl-CoA carboxylase 1, *ACC1* and fatty acid synthase, *FASN*) and reductions in the expression of lipolytic genes (hormone sensitive lipase, *LIPE* and adipose triglyceride lipase, *ATGL*) in mouse liver. These changes were accompanied by increased hepatic FTO levels and reduced m^6^A levels in mRNA [36]. Enhanced FTO expression leads to a reduction of m^6^A level and increases in the expression of lipogenic genes (*FASN*, stearoyl-CoA desaturase, *SCD* and monoacylglycerol O-acyltransferase 1, *MOGAT1*) and intracellular triglyceride level in HepG2 cells. A mutant FTO, lacking demethylase activity, fails to produce these effects [52]. Treatment with betaine, a methyl donor, increases m^6^A levels and reduces *FASN* and *SCD* mRNA levels and triglyceride levels in HepG2 cells [52]. Consistent with these findings, betaine treatment prevents high-fat diet-induced hepatic steatosis by rectifying m^6^A mRNA hypomethylation state and up-regulation of FTO and lipogenic gene expression in the liver [36]. Taken together, these findings support the possibility that hepatic FTO plays a role in the regulation of lipid metabolism by altering m^6^A modification status and expression of lipid metabolism-related genes in the liver (Figure 3).

As described above, ATF4-deficient mice are lean and resistant to high-fat diet-induced obesity. Interestingly, ATF4 deficiency or ATF4 knockdown can also protect the liver from diet-induced and ethanol-induced steatosis in mice [44,46,56,57]. Expression of lipogenic enzyme-encoding genes and production of very low-density lipoprotein (VLDL)-triglyceride is reduced in the liver of ATF4-deficient mice [47]. Enhanced ATF4 expression causes increases in lipogenic gene and protein expression and triglyceride levels in primary hepatocytes [47]. Similarly, transgenic overexpression of ATF4 results in increased lipid accumulation, leading to hepatic steatosis in zebrafish [58]. Given that FTO is a positive regulator of ATF4 expression, increased activity of the hepatic FTO-ATF4 system may promote hepatic lipid accumulation, while inhibition of this system may contribute to the reduced lipid deposition in the liver (Figure 3).

## 7. Future Perspectives

**Figure 4:**
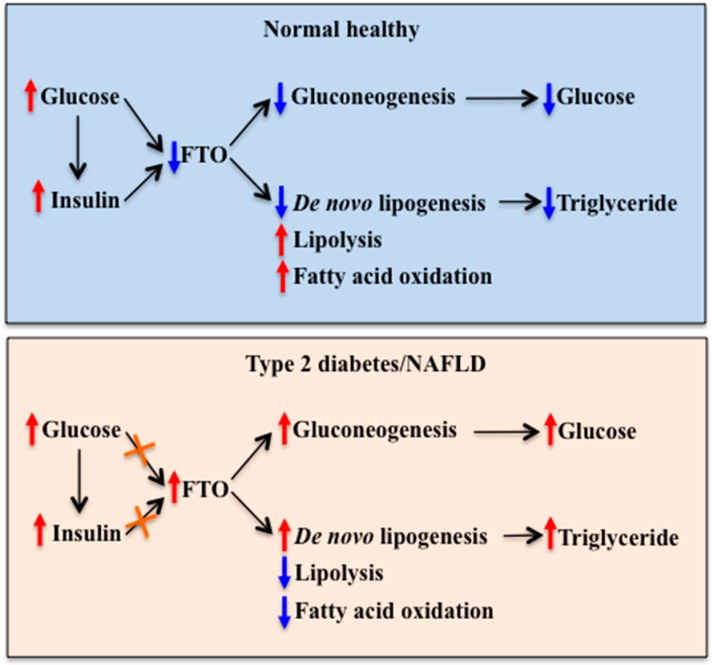
Role of FTO in the mediation of nutritional and hormonal regulation of hepatic glucose and lipid metabolism in health and diseases. Upper panel: In normal healthy individuals, increased blood glucose and insulin levels inhibit FTO expression in the liver. Reduced hepatic FTO expression inhibits gluconeogenesis, leading to reduced hepatic glucose production. It also inhibits *de novo* lipogenesis, while stimulates lipolysis and fatty acid oxidation, leading to reduced triglyceride deposition in the liver. Lower panel: In individuals with type 2 diabetes or NAFLD, impairments in glucose and insulin regulation of FTO expression may cause an increase in hepatic FTO expression. Increased hepatic FTO expression stimulates gluconeogenesis and *de novo* lipogenesis and inhibits lipolysis and fatty acid oxidation, leading to abnormally increased hepatic glucose production and triglyceride deposition. FTO: Fat mass and obesity-associated, NAFLD: Non-alcoholic fatty liver disease. Red arrow: Stimulation. Blue arrow: Inhibition. Orange cross: Impaired response.

Currently available data on the regulation of hepatic FTO expression by metabolic signals and the effect of altered hepatic FTO levels on glucose and lipid metabolism put hepatic FTO on the current map of metabolic control. It is proposed that hepatic FTO is involved in the regulation of blood glucose levels as part of a negative feedback loop. This regulation involves FTO-induced up-regulation of gluconeogenic gene expression at least partly through interactions with transcription factors. Increased blood glucose levels and glucose-induced insulin secretion may cause a reduction of hepatic FTO expression, leading to reduced gluconeogenic gene expression. Conversely, low levels of blood glucose and insulin (such as during fasting) may stimulate hepatic FTO expression, leading to the increased expression of gluconeogenic genes. Hepatic FTO is also involved in the regulation of hepatic lipid metabolism by modulating gene expression through alterations in m^6^A RNA modification status and interactions with the transcription factor. Impairments in these regulatory mechanisms may contribute to the pathogenesis of metabolic diseases such as type 2 diabetes and NAFLD, while restoration of these impairments may be beneficial in reversing metabolic abnormalities (Figure 4). Moreover, increased FTO expression in the liver may contribute to the development of type 2 diabetes and NAFLD. This raises the possibility that obesity-associated *FTO* variants may be associated with increased expression and/or activity of FTO in the liver. Further studies are necessary to determine this possibility.

If elevated FTO levels and/or activity in the liver contributes to the increased hepatic gluconeogenesis and *de novo* lipogenesis and reduced lipolysis and fatty acid oxidation, inhibition of FTO expression and/or activity may offer a novel therapeutic approach to treat diabetes and NAFLD. In a screen for compounds that inhibit the demethylase activity of FTO, several small molecules have been identified. Rhein (4,5-dihydroxyanthraquinone-2-carboxylic acid), one of the major components of *Rheum palmatum* L., has the ability to inhibit demethylase activity of FTO [59,60]. Intriguingly, rhein treatment reduces body weight and adiposity and improves glucose tolerance in high-fat diet-induced obese and insulin resistant mice [61,62]. Rhein treatment reduces *G6PC* mRNA levels in mouse hepatocyte AML12 cells [63]. These studies provide evidence in favor of beneficial effects of hepatic FTO inhibition on glucose homeostasis. Although it remains to be determined whether beneficial effects of these inhibitors are mediated by inhibition of hepatic FTO, these findings open up a new avenue towards developing therapeutic interventions through alterations in hepatic FTO activity. Knowledge of the precise role of hepatic FTO in the regulation of metabolism and its association with the pathogenesis of metabolic diseases awaits future investigations.
